# Fabrication of Self-Ordered Alumina Films with Large Interpore Distance by Janus Anodization in Citric Acid

**DOI:** 10.1038/srep39165

**Published:** 2016-12-13

**Authors:** Yingjun Ma, Yihao Wen, Juan Li, Yuxin Li, Zhiying Zhang, Chenchen Feng, Runguang Sun

**Affiliations:** 1 College of Physics and Information Technology, Shaanxi Normal University, 710119 Xi’an, P. R. China; 2 School of Science, Ningxia Medical University, 750004 Yinchuan, P. R. China

## Abstract

Self-organized porous anodic alumina (PAA) formed by electrochemical anodization have become a fundamental tool to develop various functional nanomaterials. However, it is still a great challenge to break the interpore distance (*D*_int_) limit (500 nm) by using current anodization technologies of mild anodization (MA) and hard anodization (HA). Here, we reported a new anodization mode named “Janus anodization” (JA) to controllably fabricate self-ordered PAA with large *D*_int_ at high voltage of 350–400 V. JA naturally occurs as anodizing Al foils in citric acid solution, which possessing both the characteristics of MA and HA. The process can be divided into two stages: I, slow pore nucleation stage similar to MA; II, unequilibrium self-organization process similar to HA. The as-prepared films had the highest modulus (7.0 GPa) and hardness (127.2 GPa) values compared with the alumina obtained by MA and HA. The optical studies showed that the black films have low reflectance (<10 %) in the wavelength range of 250–1500 nm and photoluminescence property. *D*_int_ can be tuned between 645–884 nm by controlling citric acid concentration or anodization voltage. JA is a potential technology to efficiently and controllably fabricate microstructured or hybrid micro- and nanostructured materials with novel properties.

Porous anodic alumina (PAA) formed by electrochemical anodization have already attracted great interest in commercial and various scientific and technological fields^1^,^2^,^3^,^4^,^5^,^6^,^7^,^8^,^9^,^10^,^11^,^12^,^13^,^14^,^15^,^16^. Due to the advantages in stability, low-cost, operating convenience and technique compatibility, electrochemical anodization of aluminum surface is employed in the industrial processing to improve its performance, such as dyeing, anti-corrosion, anti-friction^2^ and anti-dewing^3^. Moreover, the self-ordered PAA formed by the well-known mild anodization (MA) process, are not only one of the most popular templates to prepare various functional ordered nanostructures with unique optic, electric and magnetic properties^4^,^5^,^6^, but also a versatile platform to develop novel chemical and biological sensors^7^,^8^,^9^, energy storage devices^10^, drug delivery systems^11^, and so on. To facilitate various practical applications of PAA films and develop advanced functional nanomaterials, nanofabrication abilities are evolving toward precision control in the dimensions, shapes and regularity of the nanopores, which is the foundation to optimize or explore new physical, chemical and biological properties. However, in typical MA processes, hexagonally packed nanopores with fixed interpore distance (*D*_int_) of 60 nm, 100 nm and 500 nm can only be obtained^17^,^18^,^19^,^20^. To resolve this issue, HA characterized by exponential decreased current from a high value is developed to broaden the *D*_int_ range, which have recently attracted much attentions^21^,^22^,^23^,^24^,^25^. To date, highly ordered nanopore with continuously tunable *D*_int_ in the range of 70–490 nm can be realized by HA in various electrolyte systems^22^,^23^. However, it is still a great challenge to break the top limit (500 nm) to fabricate the self-ordered PAA with ultra large *D*_int_ by using current anodization technologies. As combined with other processing (e.g. nanoimprinting and electrochemical deposition), the large *D*_int_ PAA fabrication technology is very promising to become an alternative technology for traditional top-down nanofabrications^26^,^27^,^28^,^29^ to efficiently and controllably fabricate advanced materials with arrayed microstructure or hybrid micro- and nanostructure^30^ possessing novel properties and so on.

It is well accepted that *D*_int_ is closely related to the anodization voltage. For a certain electrolyte system, the higher the anodization voltage, the larger the *D*_int_^22^. Thus, the key point to fabricate highly ordered alumina film with ultra large *D*_int_ is to find a method to make the anodization can be steadily performed under a high anodization voltage. Very recently, Kikuchi *et al*. found that highly ordered porous anodic alumina with *D*_int_ between 530–670 nm could be successfully fabricated under the anodizing voltage of 210–270 V in edtidronic acid, which was a new type electrolyte possessing low acid dissociation constants. They also found the as-prepared nanostructured samples show bright structural colors and unique optical properties^31^. However, self-ordered PAA film with more larger *D*_int_ is still unreported, to the best of our knowledge. Furthermore, compared with common electrolytes (i.e. H_2_C_2_O_4_, H_2_SO_4_ and H_3_PO_4_), edtidronic acid is very expensive (US $ 200 per gram), which hinders its application. Thus, it is still significant to find a simple and low-cost way to fabricate self-ordered PAA films and explore their novel properties.

Organic carboxylic acid with larger molecular weight and lower dissociation constants, such as malonic acid, malic acid and citric acid may be a good alternative electrolyte for high-voltage anodiztion^32^. Among them, citric acid is a kind of widely used electrolyte system to fabricate PAA with large *D*_int_. To date, great efforts have been made to explore the proper electrochemical anodization conditions for citric acid, and PAA possessing large *D*_int_ greater than 500 nm can be formed by anodizing in pure citric acid solution or the mixture solution of citric acid and other electrolytes^33^,^34^,^35^. However, the obtained PAA films are disordered and the intrinsic self-ordered regime for citric acid is still unestablished by the current reported research works since anodization at higher potentials easily produce the catastrophic flow of electric current called “burning” or “breakdown events”, which causes the undesired non-uniform black oxides growth and makes the anodization cannot carry on^32^.

In this paper, we reported a new electrochemical anodization mode named “Janus anodization” to realize self-ordered PAA with ultra large *D*_int_ in the range of 675–884 nm by using critic acid solution as electrolyte. The mechanical and optical properties of as-prepared samples were also studied. By synthetically investigating the current, barrier layer thickness, film thickness and the pore arrangement evolution of the nanopores that was anodized at 400 V, we found that Janus anodization was a new process that possesses both the characteristics of MA and HA. The whole process can be divided into two stages: I, slow pore nucleation stage similar to that of MA; II, unequilibrium self-organization process similar to that of HA. The as-prepared films showed the highest modulus (7.0 GPa) and hardness (127.2 GPa) values compared with the alumina that were obtained by MA and HA in 0.3 M oxalic acid. The black films also showed low reflectance less than 10 % in the wavelength range of 250–1500 nm, and obvious photoluminescence properties (PL). *D*_int_ can be tunable in the range of 645–884 nm by controlling the electrolyte concentration or anodization voltage. These findings open a new way to explore large period PAA films, and are very helpful to develop new surface coating for aluminum and fabricate advanced chemical and biological sensors. The Janus anodization process is potential to become an alternative technology for traditional top-down nanofabrication technologies to efficiently and controllably fabricate microstructure or hybrid micro- and nanostructure with novel properties^26^,^27^,^28^,^29^,^30^.

## Results

### Characteristic of Janus anodization

**Figure 1:**
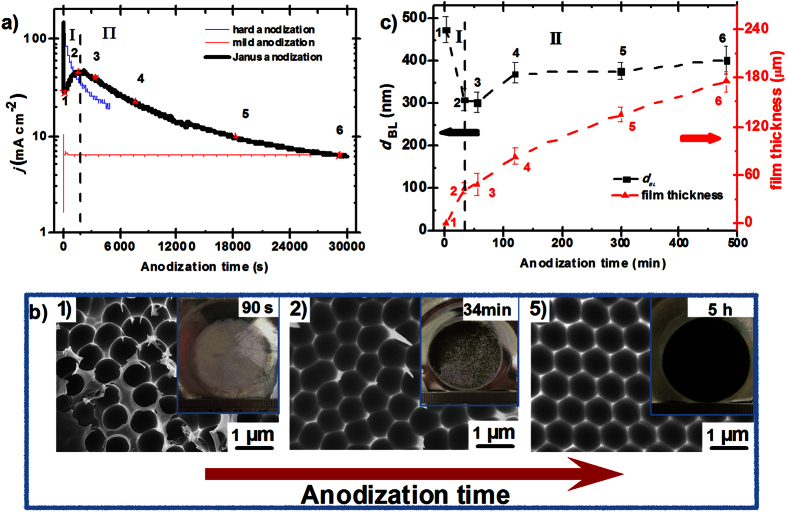
Characteristic of Janus anodization. (**a**) Current-time transients during Janus anodization of electropolished aluminum substrates in 1.5 M citric acid (0 °C) at a constant voltage of 400 V. The current-time transient of mild anodization (red line) and hard anodization (blue line) in 0.3 M oxalic acid are also plotted for comparison. Here the Arabic numbers of 1, 2, 3, 4, 5, 6 that marked on the graph stand for anodization time of 90 s, 34 min, 55 min, 2 h, 5 h and 8 h, respectively. The whole anodization process can be divided to two stages: I, pore nucleation; II, pore self-organization. (**b**) Three representive SEM images of the nanodents with varied anodization time of 90 s, 34 min and 5 h, which correspond to the Arabic number of 1, 2, 5 in a), respectively. The inserts show the corresponding macroscopic optical image of as-prepared alumina film, whose color is gradually changing from gray to black as the anodization time increase. (**c**) The barrier thickness (*d*_BL_) and film thickness varied with anodization time.

Different from previous studies in pure citric acid anodization, we found that the anodization can be stably performed at high voltage (400 V) in 1.5 M citric acid. It should be noted that a large amount of heat would be released from the reaction zone at such high anodization voltage. Here, to avoid the burning event so as to ensure stable anodization, we adopted a simple but powerful strategy, simultaneously enhance circulation cooling to both the back of Al foils and the electrolyte solution of 1.5 M citric acid, which was also used in HA to efficiently remove the reaction heat by using a home-made instrument (Figure S1). Interestingly, this stable anodization process in citric acid at high voltage naturally presented both the characteristics of MA and HA, which was defined as “Janus anodization” (JA) in this paper. Figure 1a shows the current-time transient of JA (black line), the transients of MA (red line) and HA (blue line) in 0.3 M oxalic acid are also plotted for comparison. Here the Arabic numbers of 1, 2, 3, 4, 5 and 6 marked on the graph stand for anodization time of 90s, 34 min, 55 min, 2 h, 5 h and 8 h, respectively. According to variation characteristic of the current-time transient, the transients can be divided into two parts: I, the current density (*j*) quickly decreases from the high initial value to the lowest value of 25 mA/cm^2^ (1) at the beginning and then slowly increases to the peak value of 44 mA/cm^2^ (2), which is very similar to the pore nucleation process of MA. However, it takes 34 min to accomplish this variation, which is much longer than that (<30 s) of MA. And *j* is also much higher than that of MA (<10 mA/cm^2^); II, the current shows nearly exponential decrease after 2 instead of reach a steady-state growth in MA, which is very similar to the unequilibrium self-organization process of HA, but the decrease speed is much slower than that of HA^36^. It is easy to be understood that the variation of the current is related to the morphology change of the alumina in constant voltage anodization. To have a deep insight into such novel current trend in JA, we investigated into the morphology evolution of the alumina by studying into the variation of pore arrangement, barrier thickness (*d*_BL_) and film thickness along the anodization time.

The variation of pore arrangement can be reflected by the nanodents arrangement at different anodization time, which was obtained by peeling off the corresponding alumina. Figure 1b and Figure S2 show several representative scanning electron microscope (SEM) images of nanodents with varied anodization time. The inserts show the corresponding optical image of the as-prepared alumina. It was found that the nucleation process of the nanopores in JA was very similar to that in MA. As shown in Fig. 1a and Figure S3, in stage I, pores begin to randomly appear on the surface of the alumina as *j* reach the lowest value of 90 s (1). At this time, unregularly arranged nanodents are left on certain area of the aluminum after peeling off the gray alumina, while most of the surface were flat due to the barrier layer with no pore formed (Figure S3). After that the area with nanodents become more and more larger as time increasing. As *j* gradually increases to the peak value at 34 min (2), the whole surface of the aluminum is covered by unregularly arrayed nanodents, which represents the full development of the nanopores. It should be noted that the color of the alumina gradually changes from gray to black in this process. *According to the previous studies on the anodization in citric acid,* black point on alumina surface means the undesired “burning” points, where the catastrophic rise in electronic current would make the anodization unable to proceed^32^. However, in JA, the catastrophic flow of the electronic current didn’t happen. In stage II, the arrangement of the nanodents become more and more order as the anodization time prolongs, accompanying by the color of the alumina turns to uniform black. Highly ordered nanodents with interpore distance of 884 ± 28 nm (*D*_int_) can be obtained as anodizing for 5 h.

It had been reported that deposition of anion incorporated alumina above the anion-free layer to produce an outer layer is very important for the pore nucleation process, which rate is dramatically influenced by the type of film-forming anions^37^,^38^. Pores are prefer to develop at where the deposition rate is low^37^. It is possible that citrate anion can form stable Al-citrate complexes^39^ that delay the deposition process of the outer impure alumina deposition. As the black color is caused by the incorporated carboxylate ions in the oxide film^40^,^41^, it can be say that it takes much more time for citric ions incorporation into the whole surface of the formed alumina, where only locally grown black spots appeared on it at first 90 s, and then the black color gradually covers the whole film as the anodization time prolongs.

The variation of *d*_BL_ and film thickness along anodization time was shown in Fig. 1c. It can be found that *d*_BL_ change oppositely to the current, which obey the rule that the logarithm of the current density is inversely depending on *d*_BL_^21^. As the current decreases to the lowest value (1), *d*_BL_ reach to its peak value of 475 ± 32 nm. At this point, the ratio of *d*_BL_ to voltage is ~1.2 nm/V, which is similar to that of MA. Note that, *d*_BL_ and the film thickness are the same at the point 1 because nanopores just nucleated on the alumina surface in this situation. After that, *d*_BL_ gradually decrease to 302 ± 22 nm as the current rise to the maxima (2). Then, *d*_BL_ slowly increase to 400 ± 33 nm (6) in stage II because the dissolution and the generation of the oxide approach equally to each other^36^. At this point, the ratio of *d*_BL_ to voltage is ~1 nm/V, which is similar to that of HA. The variation of the film thickness is shown in Fig. 1c. It should be noted that there are a little fluctuation about the thickness of the PAA films. For example, the thickness of the film obtained in 1.5 M citric acid electrolyte at 400 V for 5 h (0 °C) is 135 ± 8 μm. It can be calculated from Fig. 1c that the average growth rate decrease from 14 nm/s (from 1 to 3) to 8 nm/s (from 4 to 6), which is always between the MA and HA, and consistent with that of current density.

### Mechanical and optical properties of alumina films fabricated by Janus anodization

**Figure 2:**
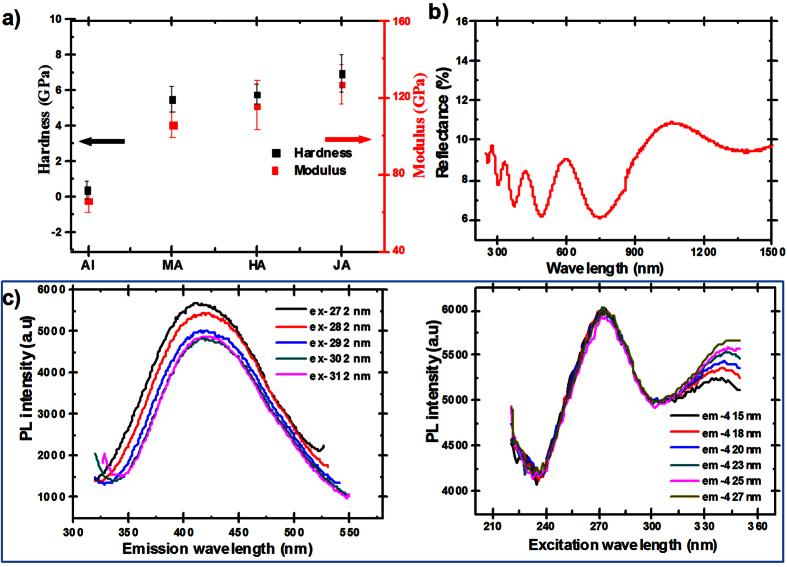
Mechanical and optical properties of alumina films fabricated by Janus anodization (JA). (**a**) Indentation modulus (7.0 GPa, red rectangular) and surface hardness (127.2 GPa, black rectangular) of JA alumina, which has the highest value in comparison with that of flat Al, MA alumina and HA alumina. (**b**) Hemispherical reflection spectra of the black JA nanosample. The average reflectance is below 10 % in the wavelength range of 250–1500 nm. Five characteristic peaks at 275 nm, 329 nm, 423 nm, 595 nm, 1061 nm appear on the spectra, which may result from interference of light reflecting from the air-porous alumina and the porous alumina-aluminum interfaces. (**c**) Photoluminescence spectra of JA alumina, the left is emission spectra and the right is excitation spectra.

It had been reported that the maxima voltage can be sustained in a steady anodization process closely related to the material ratio of formed alumina: the thicknesses of inner relatively pure alumina region to acid anion-contaminated outer region^38^. For JA, the high constant anodization voltage suggests the high ratio of relatively pure alumina in the film. Thus, it can be speculated that the as-prepared film should have excellent mechanical stability. To verify this hypothesis, we investigated into the mechanical properties of the films obtained by JA in 1.5 M citric acid at 400 V for 5 h. Modulus and surface hardness of the as-prepared film were evaluated by using the nanoindentation technique^42^. As shown in Fig. 2a, the values of the flat pure Al surface, alumina surface obtained by MA and HA were also tested for comparison. It can be seen that the mechanical stability of Al surface can be obviously improved by anodization. The alumina film obtained by Janus anodization possesses the highest hardness and modulus among the three kinds of anodic films, which increased from 0.6 GPa and 66.3 GPa of flat Al surface to 7.0 GPa and 127.2 GPa, respectively. More ever, we also found that it needed more than 10 hours to dissolve the JA alumina in the mixture solution of chromic acid and phosphoric acid at 70 °C, which was much longer than that of alumina film obtained by MA and HA (3 h). This suggests that alumina films obtained by JA also have excellent anti-corrosion ability. The mechanical property tests show that JA technique can be applied to obtain robust alumina film for practical application.

This new type film also show interesting optical properties. The black color caused by the incorporated critic acid suggests that the film should have excellent anti-reflection properties. Accordingly, we measure the hemispherical reflection (specular + diffuse) of the nanosamples, as shown in Fig. 2b. It is evident that the sample shows low reflectance in the ultraviolet, visible and near-infrared light, where the average reflectance is 9.4 % in the wavelength range of 250–1500 nm. Notably, the reflectance spectrum shows oscillation along the wavelength. There are five wave crests at 275 nm, 329 nm, 423 nm, 595 nm, 1061 nm, which may result from interference of light reflecting from the air-porous alumina and the porous alumina-aluminum interfaces^8^ (Fig. 2b). Besides, the incorporated critic acid in oxide films during the anodization can also be served as PL centres^41^. Typical PL emission (left) and corresponding excitation (right) spectra of porous oxide films are shown in Fig. 2c. Wide PL bands are presented in the wavelength ranging from 320 nm to 550 nm. The emission spectra shows that, for all measurements, there is one peak centered at a constant wavelength of about 420 nm. For the excitation spectra, one spectral peak remains at a constant wavelength of 272 nm, while the other peak shifts a little to longer wavelengths with the increasing emission wavelength. These results suggest that PL emission band with peak position around 420 nm originates from the first excitation peak 272 nm. These novel optical properties can be applied to develop new type chemical and biological sensors^7^.

### Influence of citric acid concentration

**Figure 3:**
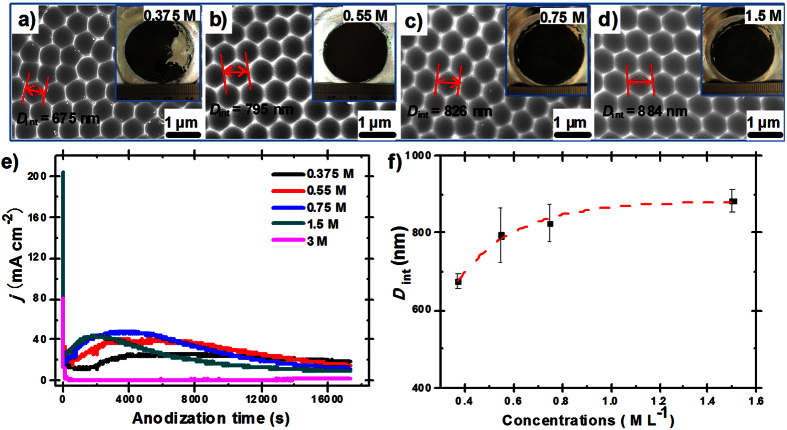
Influence of concentration. (**a–d**) SEM images of highly ordered nanodents with different interpore distances (*D*_int_) of 675 nm (**a**), 795 nm (**b**), 826 nm (**c**) and 884 nm (**d**), which were obtained by peeling off the corresponding alumina that was anodized in the electrolyte with critic acid concentration (*C*) of 0.375 M, 0.55 M, 0.75 M and 1.5 M at 400 V for 5 h, respectively. The inserts show the optical images of the alumina. (**e**) Current-time transients during Janus anodization of aluminum foils in the electrolyte with *C* increasing from 0.375 M to 3 M. Note that it takes fewer time for the current to reach the maxima as *C* increase. However, the current has not increased after reaching the lowest value as *C* increase to 3 M. (**f**) *D*_int_ varied with the concentration of critic acid as the other anodization conditions are the same, which can fit the exponential decay curve of *D*_int_ = 881.53–974.64e^−4.18*C*^.

It had been reported that for a given acid electrolyte, the concentration of the electrolyte and the electric field strength have significant influence on the anodization process^43^. As a new electrochemical anodization technology, we found *D*_int_ can also be easily tuned by either changing the citric acid concentration or anodization voltage. As shown in Fig. 3, hexagonally arranged nanodents with increased *D*_int_ from 675 nm to 884 nm can be achieved by peeling off the alumina, which was obtained by JA at 400 V for 5 h with *C* increasing from 0.375 M to 1.5 M (Fig. 3a–d). Interestingly, alumina film with two different (black and gray) parts can be fabricated as *C* = 0.375 M (Fig. 3a). It is found that unregularly nanodent regions spaciously distribute on the Al surface after peeling off the gray region (II) of the formed aluminum film, and most of the gray surface have no pore formed where nanodents cannot be found (Figure S4e). While highly ordered nanodents can be founded under the black surface of the film (Figure S4b). Similar situation can also be found as *C* increase to 3 M, where almost the whole surface of the alumina show the gray color. Although the whole surface is covered by nanopores, the growth speed of the film is very small and only unregularly nanodents can be founded after peeling off the alumina (Figure S5). To have a deep insight into this phenomenon, we investigate into the *j* variation under different concentrations. As shown in Fig. 3e, the peak value of current density (*j*_p_) gradually increase from 26.33 mA cm^−2^ to 48.54 mA cm^−2^ as *C* increase from 0.375 M to 0.75 M. And duration needed to reach *j*_p_ also decrease from 101 min to 34 min as *C* increase from 0.375 M to 1.5 M. However, out of our expectation, *j*_p_ has even not appeared as *C* further increase to 3 M. It is believed that current is mainly related to the mobile ionic (O^2−^, OH^−^, Al^3+^ and acid anions) through the barrier layer. Critic acid is a kind of strong organic acid and the amount of free anions (e.g. H^+^ and C_5_H_7_O_5_COO^−^) will increase as increase *C*, which can promote the current and simultaneously facilitate the dissolution of alumina to guarantee the homogenous nucleation of the nanopore. On the contrary, as the concentration of citrate anion further increase, stable Al-citrate complexes^39^ can form in the electrolyte, which dramatically reduce the concentration of the mobile ionic and prevent the formation of hydrated alumina^37^. Clearly, it is needed to maintain a proper *C* in Janus anodization to obtain highly ordered nanodents. Finally, we found highly ordered nanodents with varied *D*_int_ between 675 nm to 884 nm can be fabricated by adjusting *C* according to the function *D*_int_ = 881.53–974.64e^−4.18*C*^(Fig. 3f).

### Influence of anodization voltage

**Figure 4:**
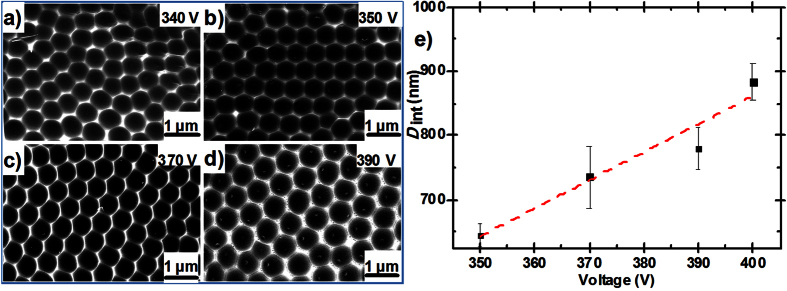
Influence of anodization voltage. (**a**–**d**) SEM images of nanodents that were obtained by peeling off the corresponding alumina which were anodize in 1.5 M citric acid at varied voltage of 340 V (**a**), 350 V (**b**), 370 V (**c**) and 390 V (**d**). High-ordered arranged nanodents cannot formed as the anodization voltage as low as 340 V. Break down events will occur as the voltage is higher than 400 V. (**e**) The evolution of interpore distance (*D*_int_) as a function of Janus anodization voltage. *D*_int_ linearly increased with the anodization voltage with a proportionality constant ζ_JA_ = 4.37 nmV^−1^.

Similarly to HA, we found there also existed a broader voltage range in JA that can obtain highly ordered nanodents with different *D*_int_. As shown in Fig. 4a, the arrangement of the nanodents images revealed that the cell homogeneity of the PAA films increases dramatically as soon as the anodization voltage is higher than 340 V. The most highly ordered nanodents with interpore distance of 645 ± 19 nm, 736 ± 48 nm, 780 ± 32 nm were obtained under 350 V, 370 V and 390 V in 1.5 M citric acid at 0 °C, respectively. According to scanning electron microscopy (SEM) analyses, the typical size of the ordered domains is in the range of 4–5 μm. Figure S6 shows the current-transient under different anodization voltages. It is found that *j*_p_ gradually increases and the time needed to reach *j*_p_ also decreases as *U* increase from 340 V to 400 V, which is very likely to the situation in concentration variation. Note that “break down” event will happen as the applied anodization voltage higher than 400 V, where the current cannot drop down in the whole anodization process. We found that *D*_int_ was also linearly proportional to the anodization voltage in JA, which can be expressed as *D*_int_ = 4.37*U*-888 (Fig. 4e). Note that the proportionality constant ζ_JA_ = 4.37 nmV^−1^ for PAA films formed by the JA process is much higher than that for the MA (ζ_MA_ = 2.5 nmV^−1^) and HA (ζ_HA_ = 2.2 nmV^−1^), which means *D*_int_ very sensitive to the anodization voltage. Thus, highly ordered alumina films with tunable *D*_int_ in the range from 645 nm to 884 nm can be obtained by changing the anodization voltage from 350 V to 400 V. Interestingly, by reducing anodization temperature or decreasing the citric acid concentration, the up limited anodization voltage can be further increased. But *D*_int_ is not increased correspondingly (Figure S6). The electrochemical parameters influencing the process of JA is still needed to be further investigated.

## Discussion

We found that the anodization in pure citric acid can be stably performed at ultra-high voltage by simply removing the reaction heat from both the aluminum surface and electrolyte, which is a new anodization mode named “Janus anodization” and present distinct features as below: (1) MA-like pore nucleation process but high current density and take much more longer time; (2) HA-like self-organization process but moderate decline of the current density; (3) the black film is the product of Janus anodization, which demonstrates good mechanically stability, anti-corrosion ability, low reflectance in the wavelength range of 250–1500 nm and PL property; (4) highly ordered nanodents with large *D*_int_ between 645 nm to 884 nm can be obtained by adjusting the electrolyte concentration or anodization voltage. The reasons that result in these interesting phenomena may relate to the dissociation properties of the citric acid and the reaction between citrate and aluminum ion. However, the formation mechanisms of these phenomena are still unclear, and the electrochemical parameters influencing the process of Janus anodization still need to be further investigated.

It should be pointed out that Janus anodization may be also suitable for other organic electrolytes with larger molecular weight, which opens a way to explore self-ordered PAA films with even larger interpore distance. Due to the excellent mechanical stability and anti-corrosion ability of the as-prepared film, JA is very promising to evolve into a practical manufacture technique to process new coating on aluminum surface. Moreover, JA only needs very simple and inexpensive apparatuses, which are accessible for common researchers to controllable fabrication of micro or sub-micro arrays that are made up of metal, inorganic and polymer materials. Thus, it is potential to become an alternative technology for traditional top-down nanofabrication technologies to explore novel physical, chemical and biological properties of nanomaterials with large period arrays or hybrid micro and nanostructure^26^,^27^,^28^,^29^,^30^.

## Methods

### Fabrication of Self-Ordered Alumina Films with Large Interpore Distance by Janus anodization

Highly pure (99.999 %) aluminum disk was respectively ultrasonic cleanned in acetone and ethanol for 5 min to remove organic contaminants, and then electropolished in a mixture of perchloric acid and ethanol (V/V = 1:4) for 11 min (20 V, 0 °C). After rinsing by the deionized water for three times, the electro-polished disk was placed in a custom-tailored electrochemical cell equipped with a circle cooling system (DC-3006, Ningbo Scientz Biotechnology Co., Ltd), where the cooling liquid (ethanol) can remove the reaction heat from both the back of Al foils and electrolyte. The circle reaction zone exposed to the electrolyte was 25.4 mm in diameter. A platinum electrode was employed as a counter electrode. Citric acid solution with concentration ranging from 0.375 M to 3 M was used as the electrolyte, which was under violent agitation. Janus anodization was proceed at the target voltage from 340–450 V at 0–(−3) °C for 90 s-8 h. Current densities (*j*) were calculated by dividing the current values measured from the power supply system (IT 6726 V, ITECH) by the anodized sample area. After the anodization, the as-prepared nanosamples were immersed into a mixed solution of 1.8 wt% CrO_3_ and 6 wt% H_3_PO_4_ for 12 h at 70 °C to peel off the alumina films to obtain the corresponding nanodents.

### Characterizations

The geometrical morphologies of all samples were observed under a field-emission scanning electron microscope (FE-SEM, Nova NanoSEM 450, FEI) after sputtering a 15-nm thickness of Au layer. To obtain the morphology parameters of the nanopores, we generally observed five batches of samples and twenty pores per sample under high-resolution scanning electronic microscope. The mechanical properties of the as-prepared PAA film were characterized by nanoindentation system (Nano indenter G200 Agilent Technologies Inc., Santa Clara, CA, USA). Appling the Oliver and Pharr method^42^ to the indentations with the maximum penetration depth of 1500 nm to determine the modulus and hardness. Each sample was measured more than 20 indentations to make an average value. The hemispherical reflection spectra were measured by a spectrophotometer (Perkin Elmer lambda 950, Japan) equipped with an integrating sphere for wavelengths of 250–1500 nm. The reported reflectance is the average values of five data obtained from five spots of identical samples. PL spectral measurements were taken on a Horiba Jobin Yvon Fluorolog F-4500FL spectrofluorometer with a Xe lamp as the excitation light source at room temperature.

## Additional Information

**How to cite this article:** Ma, Y. *et al*. Fabrication of Self-Ordered Alumina Films with Large Interpore Distance by Janus Anodization in Citric Acid. *Sci. Rep.* **6**, 39165; doi: 10.1038/srep39165 (2016).

**Publisher's note:** Springer Nature remains neutral with regard to jurisdictional claims in published maps and institutional affiliations.

## Supplementary Material

Supplementary Information
