# Supplementary material for: Fabrication of Self-Ordered Alumina Films with Large Interpore Distance by Janus Anodization in Citric Acid
Source: Sci Rep. 2016 Dec 13;6:39165. doi: 10.1038/srep39165 (PMC5153624; doi:10.1038/srep39165)
Supplement: Supplementary Information [file srep39165-s1.doc]

**Supplementary Information**

Fabrication of Self-Ordered Alumina Films with Large Interpore Distance by Janus Anodization in Citric Acid

Yingjun Ma^1,2^, Yihao Wen^1^, Juan Li^1,*^, Yuxin Li^1^, Zhiying Zhang^1^, Chenchen Feng^1^, Runguang Sun^1,*^


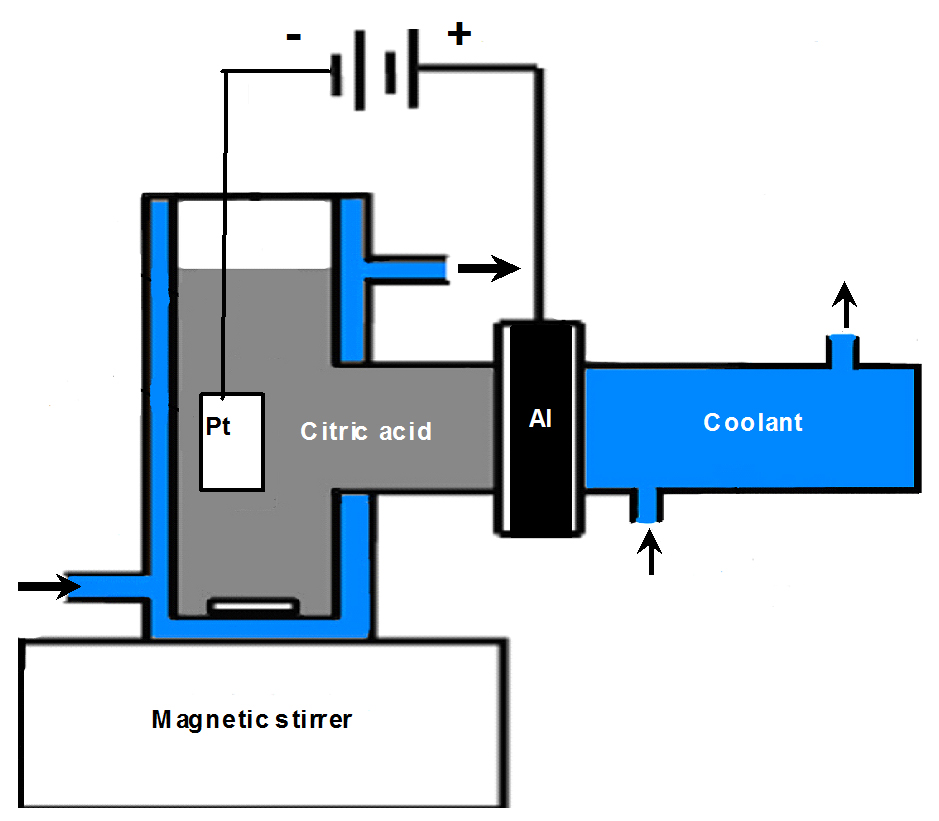


Supplementary Figure 1. Schematic model of anodization set-up.


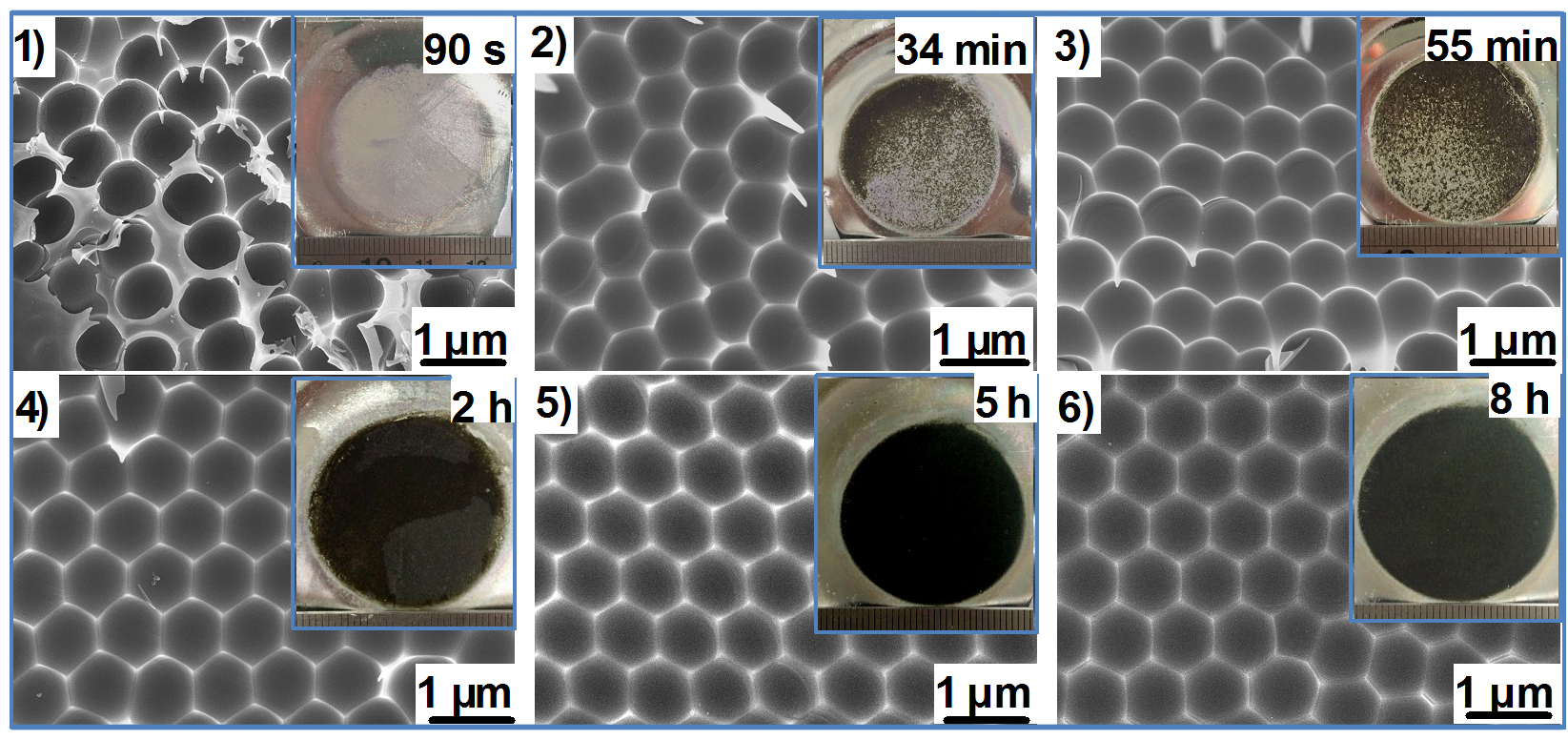


**Supplementary Figure 2.** SEM images of nanodents that were obtained by peeling off the corresponding alumina which were anodized in 1.5 M citric acid at 400 V (0 ºC) with varied time of 90 s, 34 min, 55 min, 2 h, 5 h and 8 h, respectively. The optical image on the top-right of each panel is the corresponding alumina, whose color is gradually turning from gray to black. As the anodization is 90 s, the regions with unregularly arranged nanodents randomly distributed on the aluminum surface after peeling off the PAA film, which means the nanopore just beginning to nucleate. As anodization time extends to 34 min, the whole surface of the aluminum is covered with unregularly arranged nanodents, which stand for the full development of the nanopores. With anodization time further extending, the arrangement of the nanodents become more and more order.


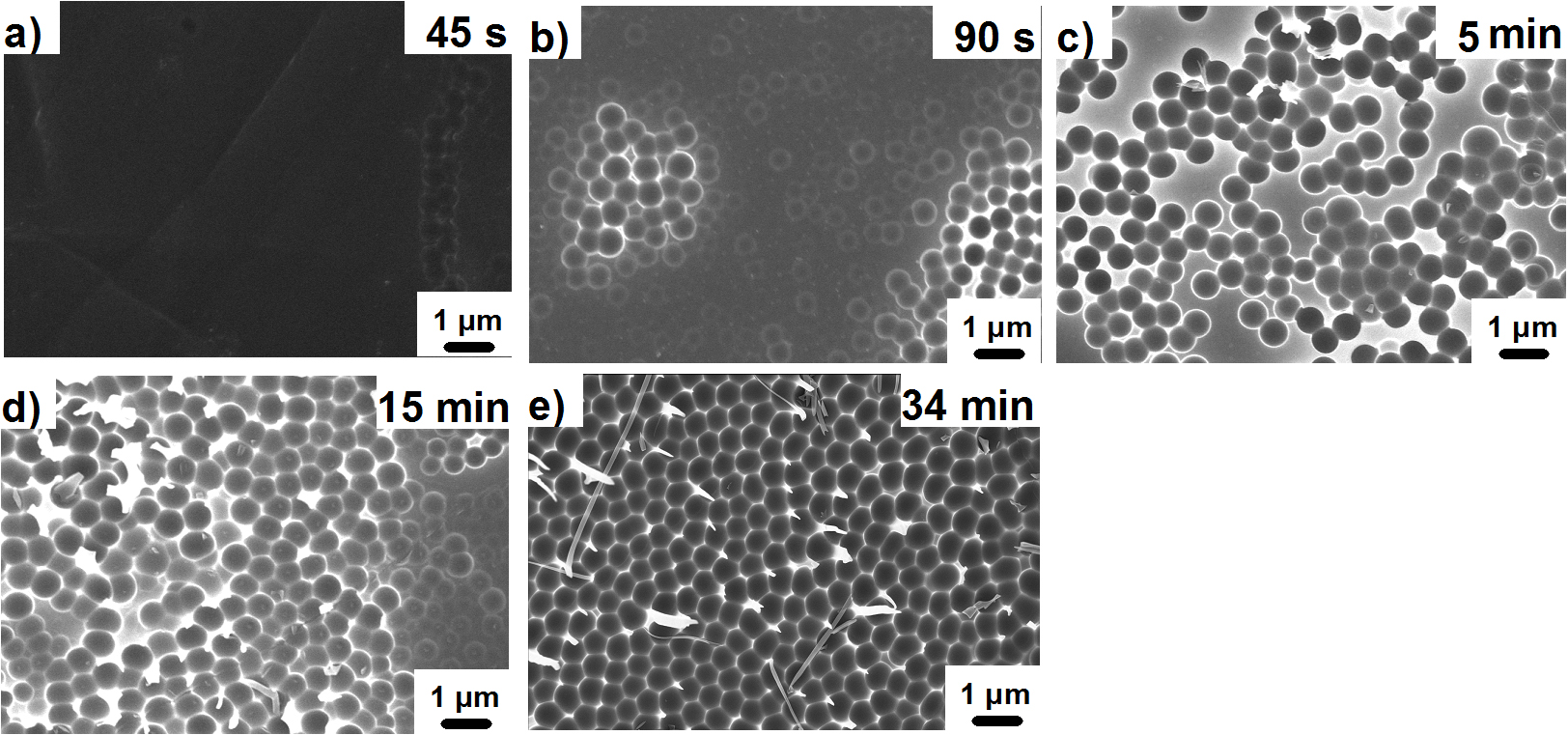


**Supplementary Figure 3.**  SEM images of the nanodents by peeling off the corresponding alumina films that were anodized in 1.5 M citric acid at 400V under different times of (a) 45 s, (b) 90 s, (c) 5 min, (d) 15 min and (e) 34 min, respectively.


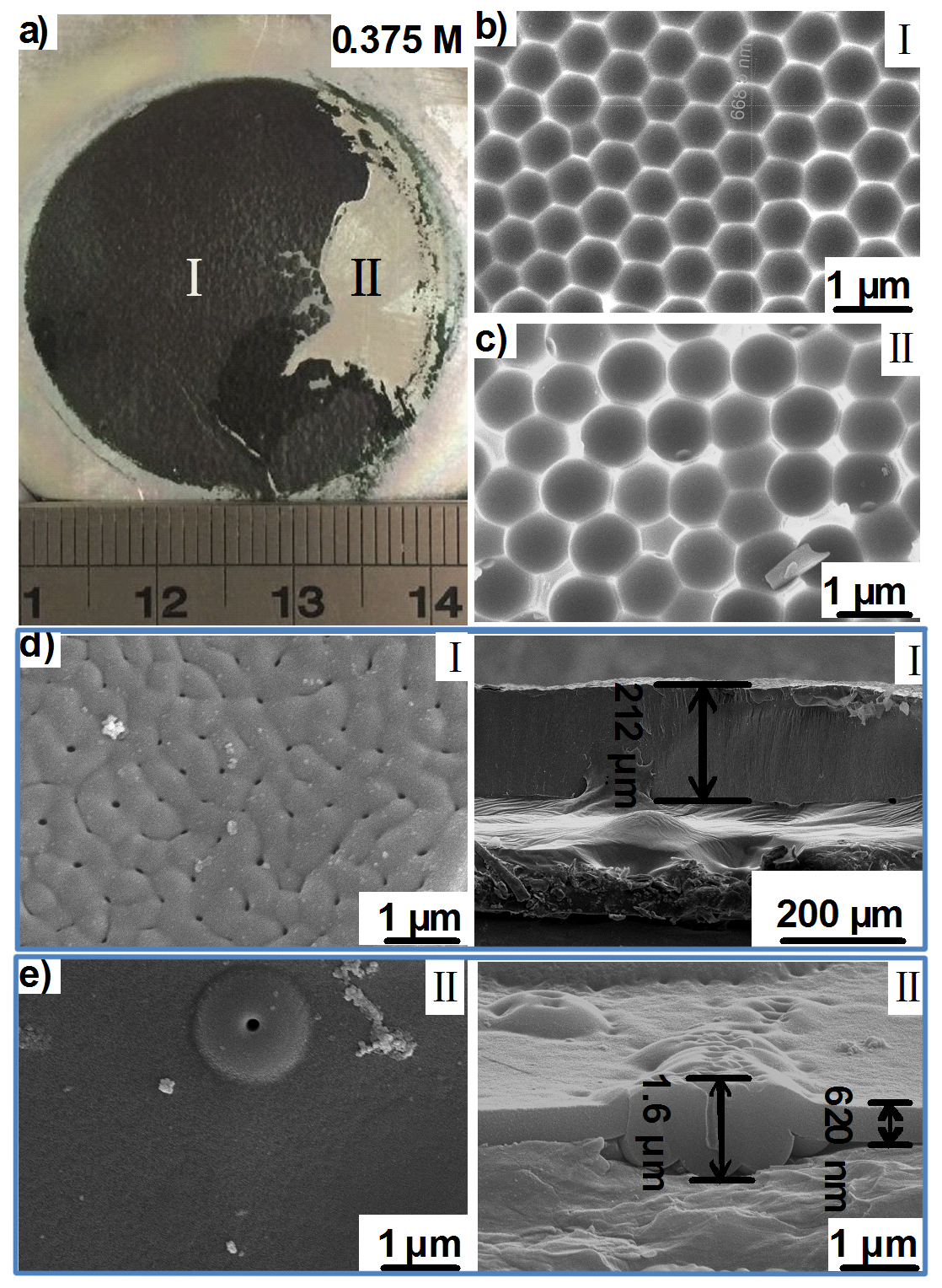


**Supplementary Figure 4.** (a) Optical image of the alumina that was obtained in 0.375 M citric acid at 400 V for 5 h. It has two obviously different regions：I black region; Ⅱ gray region. (b, c) SEM images of nanodents which are obtained by peeling off the region I (b) and region II (c), respectively. It was found that unregularly arranged nanodents spaciously distribute on the Al surface after peeling off the gray regionⅡ, while highly ordered nanodents can be founded under the black region I. d-e) SEM top-views and side-views of the alumina film formed at region I (d) and region II (e), respectively. Region I is uniformly covered by nanopores and with film thickness of 212 µm, while few nanopores are formed on region II and the pore thickness is only 1.6 µm. The film thickness at region II without nanopore is only 620 nm, though it is also anodized for 5 h.


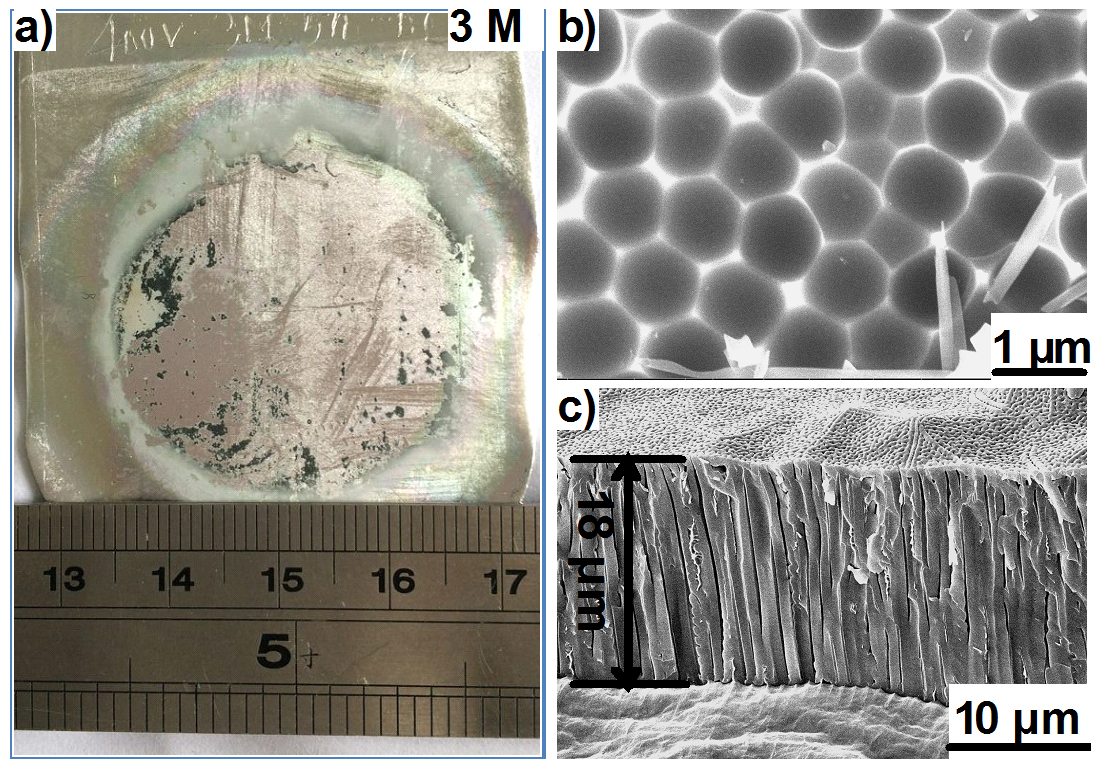


**Supplementary Figure 5.** (a) Optical image of the alumina that was obtained by anodizing in 3 M citric acid at 400 V (0 ºC) for 5 h, the whole surface of the alumina show the gray color with little black points. (b) SEM images of unregularly arranged nanodents that were obtained by peeling off the corresponding alumina. (c) SEM side-view of the PAA film with average height of 18 μm, which means the growth speed of the nanopore is very slow.


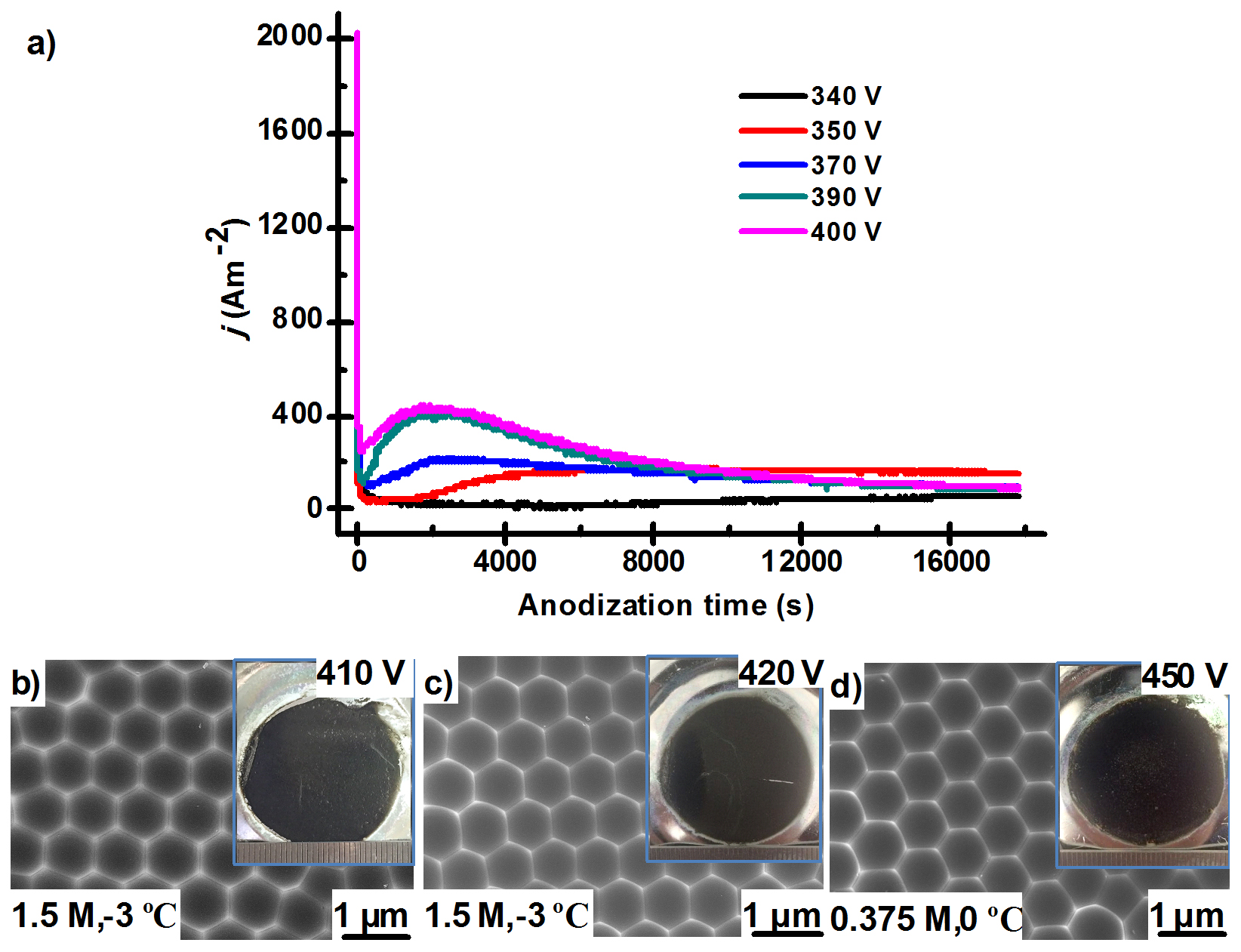


**Supplementary Figure 6.** (a) Current-time transients during the anodization of aluminum foils in 1.5 M citric acid at different potentials from 340 V to 400 V for 5 h (0 ºC), the higher voltage, the earlier current density reaches the maximum value. (b-d) SEM images of nanodents that were obtained by peeling off the corresponding alumina which were anodized in citric acid at varied voltage of 410 V(1.5 M, -3 ºC), 420 V(1.5 M, -3 ºC) and 450 V (0.375 M, 0 ºC) for 5 h, respectively. The interpore distances are 778±43 nm(b), 842±53 nm(c), and 904±30 nm(d), respectively. By reducing anodization temperature or decreasing the citric acid concentration, the up limited anodization voltage can be further increased. But *D*_int_ is not increasing correspondingly.
